# Epidemiology of maxillofacial injuries in “Heratsi” No 1 university hospital in Yerevan, Armenia: a retrospective study

**DOI:** 10.1186/s12903-022-02158-6

**Published:** 2022-04-12

**Authors:** Lusine V. Aleksanyan, Anna Yu Poghosyan, Martin S. Misakyan, Armen M. Minasyan, Aren Yu Bablumyan, Artashes E. Tadevosyan, Armen A. Muradyan

**Affiliations:** 1 Department of ENT and Maxillofacial Surgery, Yerevan State Medical University, “Heratsi” No 1 Hospital, 60 Abovyan Str., 0025 Yerevan, Armenia; 2 Administrative Department, Yerevan State Medical University, 2 Koryun Str., 0025 Yerevan, Armenia; 3 Department of Public Health and Healthcare, Yerevan State Medical University, 2 Koryun Str., 0025 Yerevan, Armenia

**Keywords:** Epidemiology, Maxillofacial fracture, Mandible, Etiology, Interpersonal violence

## Abstract

**Background:**

The aim of this study was to perform a retrospective analysis of the prevalence, etiologies, types of maxillofacial injuries (MFIs), sites of maxillofacial fractures (MFFs) and their management in Yerevan, Armenia.

**Methods:**

A retrospective cross-sectional study was conducted. The extracted data included age, sex, date of referral, mode of injury, etiology, radiology records and treatment methods. Study outcomes were measured using percentages, means, standard deviations and tests of proportions. *P* < .05 was considered significant.

**Results:**

A total of 204 patients had a mean age of 36.26 ± 1.08 years (156 males and 48 females), and a total of 259 MFIs were recorded between 2017 and 2020. Interpersonal violence was found to be the most common etiology of MFFs in this study (42.1%), followed by road traffic accidents (RTAs) (27.9%) and falls (18.6%). The nasal bone was the most common injury site (47.5%), followed by the mandible (31.4%) and zygomatic complex (11.7%). The most common fracture site was the mandibular angle (37.9%), followed by the symphysis/parasymphysis (28.1%) and body (12.6%). Isolated soft tissue injuries were reported in 5.9% of the cases. The majority of MFFs were treated by open reduction and internal fixation.

**Conclusion:**

Interpersonal violence, followed by RTAs and falls, was the most common cause of MFIs. Males in the 21–30 years age group had the highest MFI incidence rate. The nasal bone was the most common injury site, followed by the mandible and zygomatic complex. Social education with the objective of reducing aggression and interpersonal conflict should be improved, and appropriate RTA prevention strategies should be strengthened and implemented.

## Introduction

Traumatic injuries continue to be important causes of morbidity and mortality in both developed and developing regions [1–4]. The epidemiology of facial injuries varies among different countries and geographic zones. Population concentration, lifestyle, cultural background, and socioeconomic status can affect the prevalence of maxillofacial injuries (MFIs) [4–7]. In addition to population and societal changes, the incidence rates and patterns of maxillofacial fractures (MFFs) may also vary among time periods due to legislative changes such as the introduction of compulsory safety belt legislation, helmet use, and speed limit enforcement [6–10]. Traumatic injuries represent a significant and growing disease burden in the developing world and are now one of the leading causes of death in economically active adults in many low- and middle-income countries [4, 9, 11]. According to the World Health Organization (WHO), middle-income countries have higher injury and death rates than low- and high-income countries [3, 12]. In addition to an increasing total proportion of injuries in developing countries, among the total number of injuries to the maxillofacial region, the percentage of combined injuries is increasing, which indicates serious suffering among patients and prolonged hospitalization and rehabilitation [2, 13–15].

In this regard, many studies of the etiology and prevalence of injuries have been carried out all over the world, but there is still no final geographical distribution of injuries and their patterns depending on the level of development of the country and the peculiarities of the legislative order [1–12]. There are currently no studies related to the etiology of facial skeleton injuries in our region. In this regard, knowledge about the prevalence and etiology of MFI in Armenia could fulfill public health with necessary information. Knowledge about the epidemiology of MFF can help practitioners make appropriate clinical decisions and guide professionals and policy makers concerned with developing suitable injury prevention strategies.

The aim of this study was to perform a retrospective analysis of the prevalence, etiologies, types of maxillofacial injuries, sites of maxillofacial fractures and their management in the Department of ENT and Maxillofacial Surgery of <<Heratsi>> No. 1 University Hospital in Yerevan, Armenia and to suggest strategies for their prevention.

## Material and methods

This research was conducted in accordance with relevant ethical standards, and the study protocol was approved by the Yerevan State Medical University Ethics Committee [IRB №5-3/2021]. All methods were carried out in accordance with relevant guidelines and regulations.

A retrospective cross-sectional study was conducted. The medical records of hospitalized patients with MFIs admitted to the Department of ENT and Maxillofacial Surgery at <<Heratsi>> No. 1 University Hospital in Yerevan, Armenia, between January 2017 and December 2020 were retrieved and analyzed to obtain prevalence, etiology, injury pattern and treatment modality data. The study size was due to the period of time in which the data were collected (4 years).

All patients included in the study signed an informed consent form at the time of their admission to the hospital with agreement of the use of their anonymized medical data for scientific research purposes. The inclusion criteria were as follows: (1) patients with a history of acute trauma; (2) patients with soft tissue injury, which required hospitalization; (3) patients with at least one fracture line in the facial skeleton; (4) the presence of radiological examinations describing the location and characteristics of fractures; and (5) treatment of the injuries performed in the study host institution.

The exclusion criteria were as follows: (1) outpatients offered immediate treatment without hospitalization; (2) patients with only soft tissue injuries who were treated in the emergency room without hospitalization; (3) patients with pathological fractures; (4) military patients wounded during the war from October–November 2020; or (5) patients with incomplete medical record sheets. Only hospitalized patients were included in the study, as outpatient patients were treated both in the Emergency Room and in the Maxillofacial Surgery Department; thus, the statistical analyses could not be corrected. Pathological fracture could not be related to injury and could be under any masticatory forces. War and martial law is not a standard situation, and thus, military patients were also excluded from the study. Follow-up was not considered in the inclusion/exclusion criteria in the present study.

After excluding such patients, the records of 204 patients aged between 12 and 90 years were retrospectively analyzed. The sample size calculation n = Z^2^pq/ Δ^2^ was performed for a one-group proportion, where p = 0.5, Δ = 0.07, and n = 196.

Data on age, sex, date of referral, mode of injury, etiology, radiographic findings with radiology records and treatment methods were extracted. Injury etiology was classified into four main categories: (1) RTAs involving automobiles, motorcycles and bicycles, including drivers, pillion riders, passengers, and pedestrians; (2) falls from heights, household falls, and falls due to systemic illness such as epilepsy or while playing; (3) assaults or interpersonal violence; and (4) sport-related and other injuries.

The type of MFF was classified according to the following maxillofacial anatomical sites: nasal, Le-Fort, zygomatic complex, orbital floor and mandibular (subclassified into symphysis/parasymphysis, body, angle, ramus, condylar and coronoid process) fractures.

MFIs were treated with the following methods: (1) closed reduction; (2) open surgical treatment or open reduction and internal fixation (ORIF), conservative treatment and wound debridement.

Data collection tools consisted of observation and census sampling of medical records and documents. To prevent bias, all observation records were checked twice by the authors who collected the data.

### Ethical considerations

Ethical considerations were taken into account throughout the study, and the patients’ names and medical information were kept completely confidential. The subjects’ medical history was used solely for the purposes of the current study.

### Statistical analysis

Statistical analysis was performed using SPSS version 16.0 (SPSS Inc., Chicago, IL, USA). Study outcomes were measured using percentages, means, standard deviations and tests of proportions. The prevalence rates of injuries in particular age, sex, etiology, and fracture type groups were analyzed. A nonparametric statistical test (Pearson’s χ^2^) was used to analyze nominal data. Patients’ ages were expressed as the mean and SD. Patient- and injury-related variables, including age, sex, anatomic location of the fracture, and etiology, were analyzed with χ^2^ tests or tables larger than 2 × 2, and a post hoc test with Bonferroni correction was used.

## Results

**Fig. 1:**
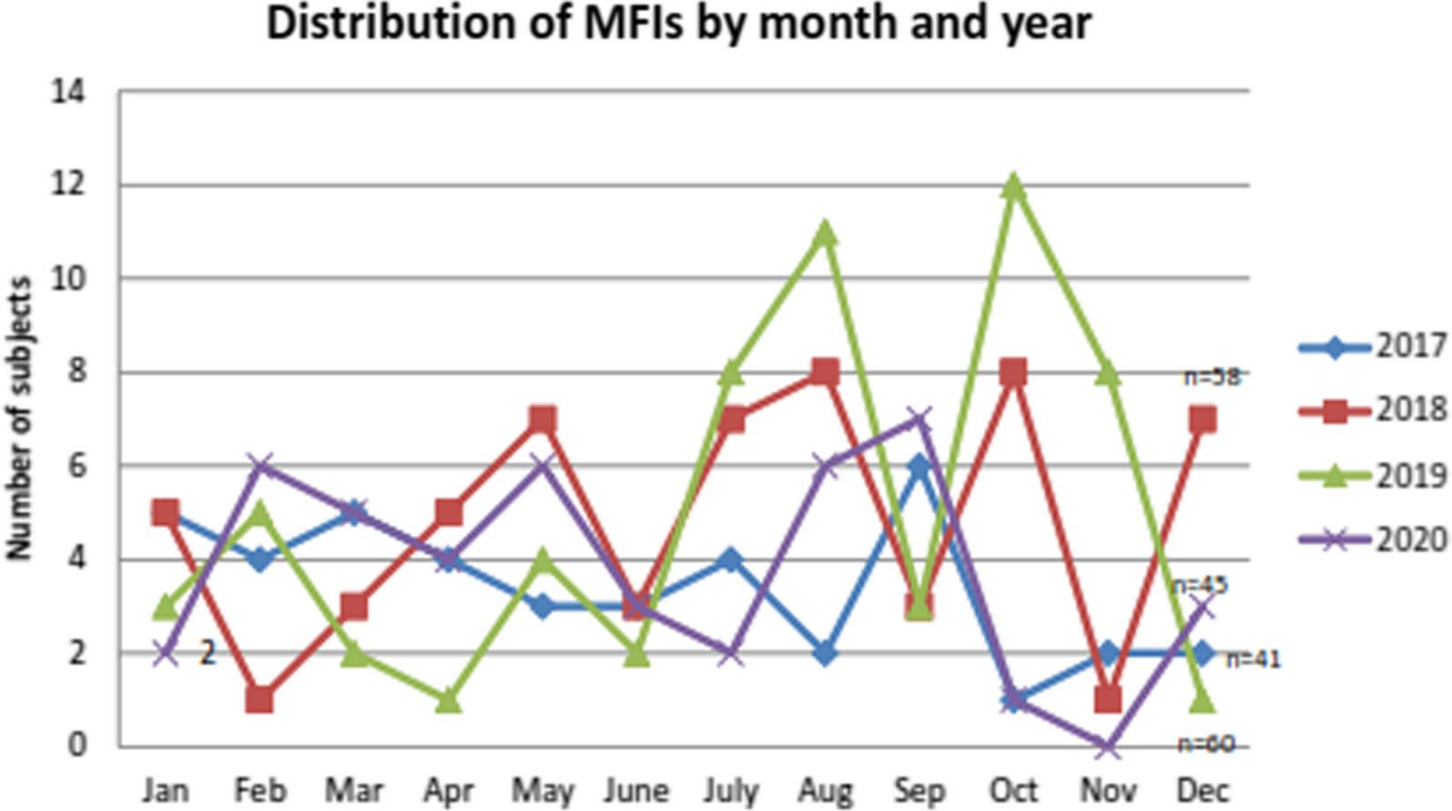
Distribution of MFIs by month and year

From 2017 to 2019, MFIs increased annually (Fig. 1). In 2020, the total number of injuries decreased because of some restrictions and lockdowns in Armenia due to the coronavirus disease 2019 (COVID-19) pandemic. Despite the absence of strict patterns, it was observed that the highest rate of fractures occurred from July to October (Fig. 1).

A total of 204 patients with 259 MFIs presented to the ENT and Maxillofacial Surgery Department between 2017 and 2020.

Patients with MFFs accounted for 190 of the 204 patients (93.14%), with a total of 242 fractures.

The mean age and standard deviation of the patients with MFIs was 36.26 ± 1.08 years, with a minimum age of 12 years and a maximum age of 90 years. Adults aged between 21 and 40 years had the highest rate. In this study, 76.5% (156/204) of the subjects were male, and 23.5% (48/204) were female, with a male to female ratio of 3:1. The test of the proportion of males and females showed that there was a significantly higher proportion of males with maxillofacial trauma (*P* = 0.0009, n = 204, χ^2^ test).

**Fig. 2:**
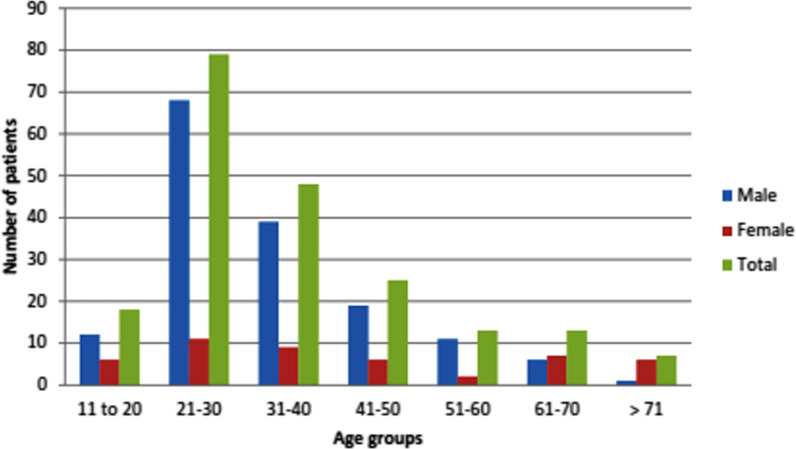
Distributions of patients by age and sex

As shown in Fig. 2, males in the 21–30 years age group had the highest prevalence (33.4%; n = 68). The highest prevalence in females occurred in the age groups over 61 years (6.4%; n = 13).

**Fig. 3:**
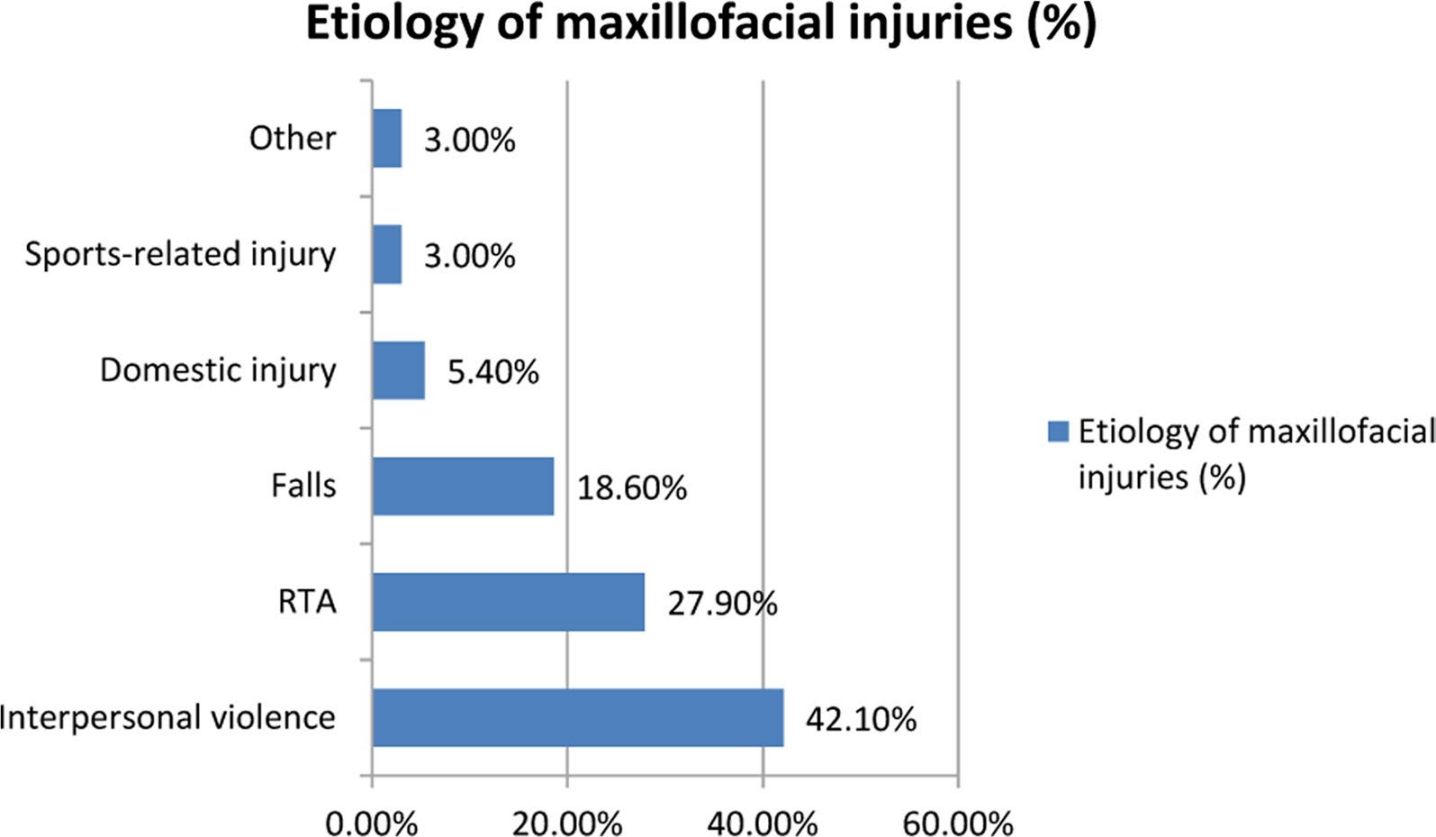
Distribution of MFIs by etiology

The most common cause of MFI was interpersonal violence (IV), accounting for 42.1% of all injuries (86/204), and male predominance was observed (94.1% vs. 1.1%; *p* = 0.0006, n = 204, χ^2^ test). RTAs accounted for 27.9% (57/204) of all injuries (Fig. 3). Car accidents and pedestrian accidents were the main causes of RTA injuries, and only one motorcycle accident was reported. Trauma due to falls accounted for 18.6% of the injuries (38/204), mostly involving females (52.6%; 20/38), elderly people who fell due to systemic illness and men who fell from heights. Domestic injuries accounted for 5.4% of all injuries (11/204). Six sports-related injuries, five industrial injuries and one suicide-related injury were reported.

**Fig. 4:**
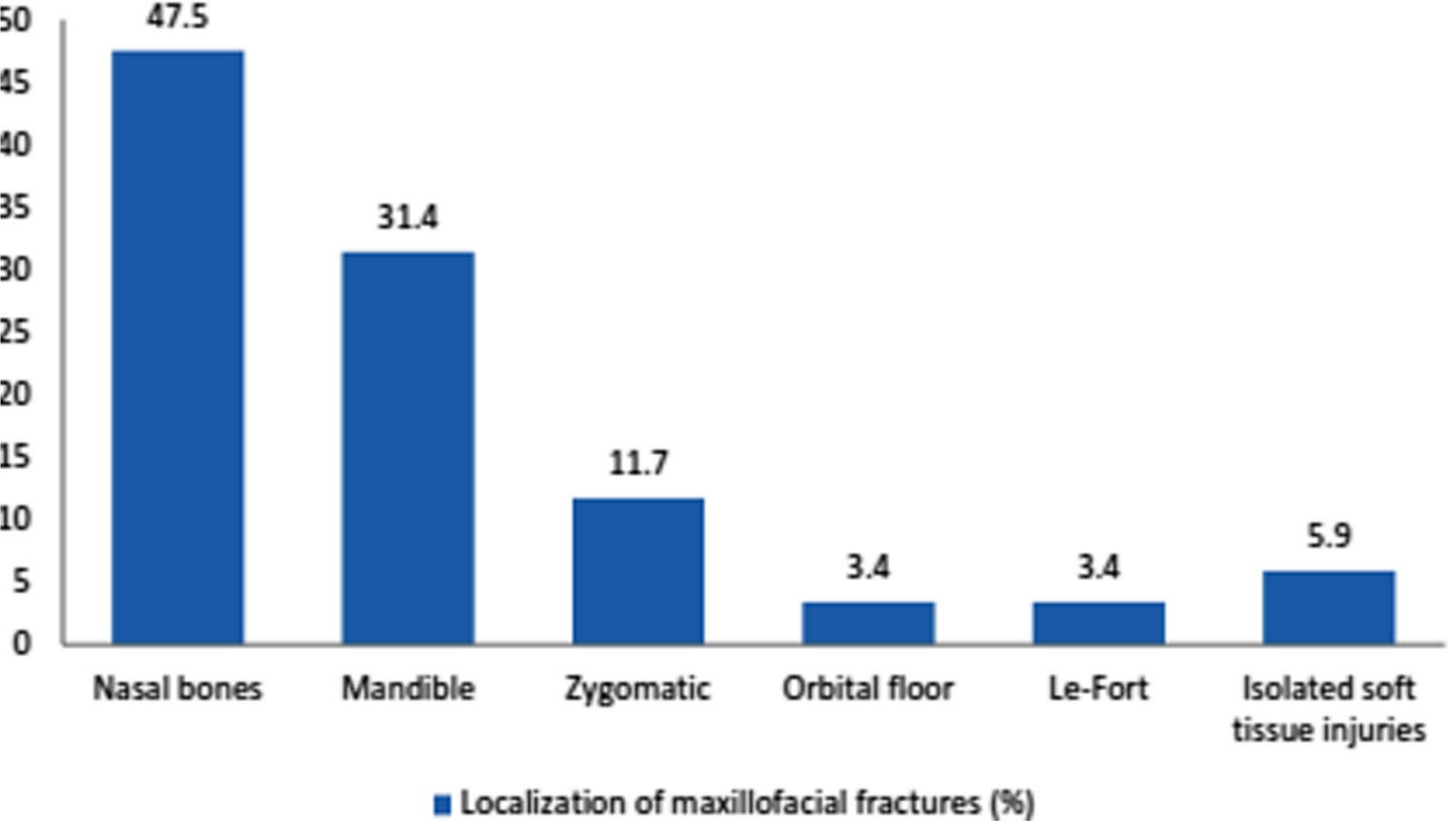
Localization of MFFs

Data analysis showed that the largest percentage of fractures occurred in the nasal bones, accounting for 47.5% of all MFIs (n = 204), of which 82 were isolated fractures of the nose and 15 were combined with other maxillofacial fractures (Fig. 4).

**Fig. 5:**
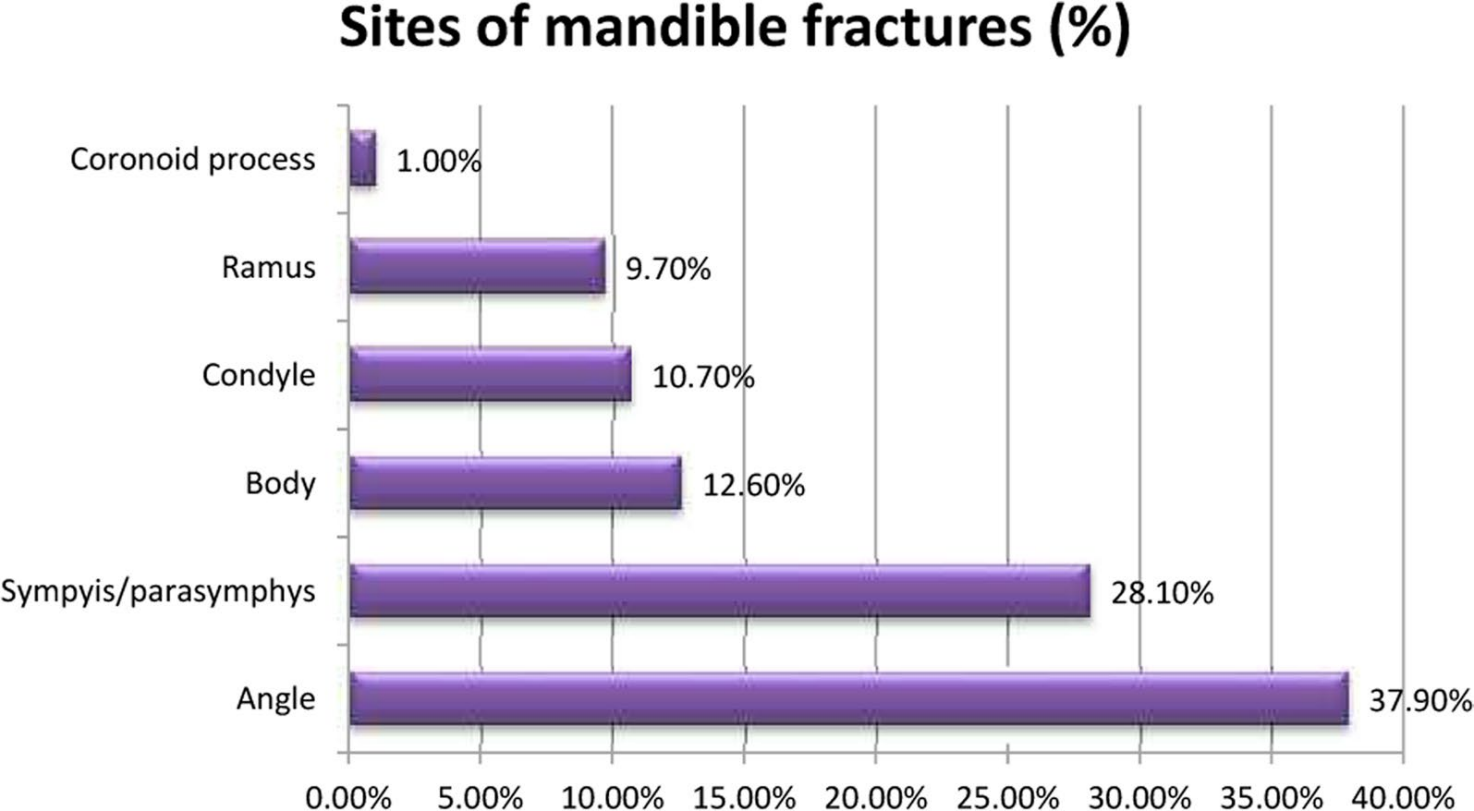
Sites of mandible fractures

Mandible fractures accounted for 31.4% (64/204) of all MFIs, and the majority of them (71.9%; 46/64) were caused by interpersonal violence (*P* = 0.011, n = 64, χ^2^ test). A sex comparison showed a significantly higher prevalence of mandible fractures in males (88.9% vs. 11.1%; *P* = 0.0052, n = 64, χ^2^ test). Bilateral fractures of the mandible were observed in 60.9% of the patients (39/64), and unilateral fractures were observed in 37.5% of the patients (24/64). The total number of mandible fracture sites was 103. The most frequent injury location of mandible fractures was the angle (37.9%), followed by the symphysis/parasymphysis (28.1%) and the body (12.6%). Condyle fractures accounted for only 10.7% of mandible fractures (Fig. 5).

Zygomatic fractures (zygomatico-orbital, zygomatico-maxillary, zygomatico-ethmoidal) accounted for 11.7% (24/204).

Le-Fort fractures were reported in seven (3.4%) cases, of which six were due to RTAs and one was due to an industrial injury. Orbital floor fractures accounted for 3.4% (7/204) of the total number of injuries. Isolated soft tissue injuries were reported in 5.9% (12/204) of the cases (Fig. 4).

Combined craniomaxillofacial trauma was observed in 7.8% of the injuries (16/204).

**Fig. 6:**
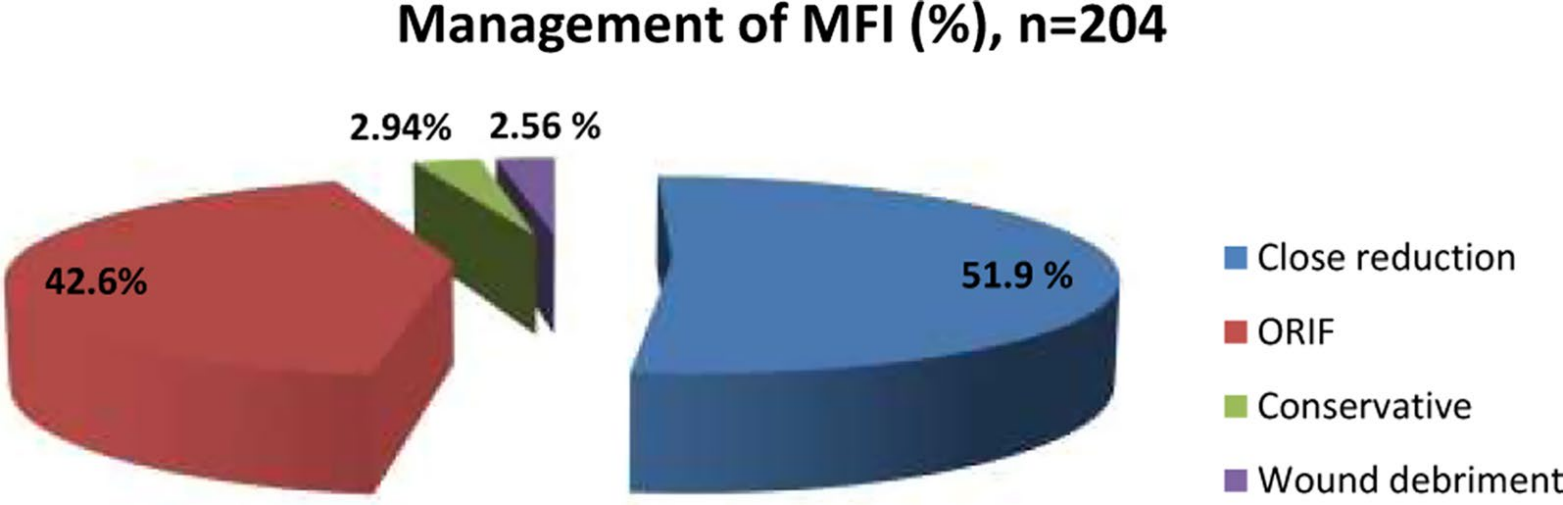
Management of MFI

A total of 2.94% of the fractures (6/204) were treated conservatively. Close reduction accounted for 51.9% of fracture treatments (106/204), 96 of which comprised repositioning of the nasal bones and 10 of which comprised close reduction of the zygomatico-maxillary fracture and zygomatic arch. A total of 42.6% of the fractures (87/204) were treated by ORIF, of which 62 were mandible fractures and 25 were mid-face fractures (zygomatic and Le-Fort fractures) (Fig. 6).

## Discussion

Maxillofacial fractures (MFFs) not only cause serious physiological injuries but also impose serious burdens on society due to morbidity, mortality, facial disfigurement, loss of function, and financial expenditures associated with such injuries [4, 16–18]. The incidence rates, etiologies, types, and injuries associated with MFFs vary among different countries and even different areas within the same country due to environmental, socioeconomic, cultural, and lifestyle differences among people [4, 7, 16]. The present study shows a high incidence of maxillofacial fractures in the 20–41 age groups, with the high prevalence of interpersonal violence as a common cause.

The proportion of males affected by MFFs in this study was higher than that of females, which is in agreement with findings reported in most other studies [1, 2, 4, 9, 19–22]. Armenian lifestyle and culture confine men and women to their traditional social roles to a certain extent. Women are rarely involved in interpersonal violence, and female drivers make up less than 2% of the total number of drivers. However, it should be noted that the percentage of women among pedestrians is higher than that of men. This is because women more often and inattentively cross the streets in prohibited places.

IV was found to be the most common etiology of MFF in this study, followed by RTAs and falls. Most studies on the etiology of maxillofacial trauma in developing countries indicate that RTAs are the most frequent cause of MFIs [3, 5, 6, 10, 17]. In contrast, the most frequent cause of MFFs in developed countries is IV or assault [1, 6, 10, 15, 23–26]. The MFF epidemiology data obtained in the present study are comparable with data from Europe and the United States [1, 24, 26]. The high level of IV in the present study could be the result of country socioeconomic factors (social, cultural and economic). The root causes of violence and the high level of criminal offenses among men vary from traditional cultural stereotypes and mentality to mistrust in law-enforcement institutions.

The 21–40 years age group had the highest MFI incidence rate in the present study. These data are in accordance with data obtained by many other researchers [6, 7, 9, 15, 17, 19, 23, 27]. The main etiological cause of injuries in the 21–30 years age group was IV, followed by RTAs. The high rate in this age group may be due to participation in outdoor activities or psychosocial problems that may provoke risk-taking behaviors, thus making this population more prone to injuries [28]. In this study, patient age was found to be associated with fracture site. It was demonstrated that patients aged 21–30 years were likely to have sustained nasal bone fractures and mandible fractures in equal proportions. The nose and the mandible are the most prominent features of the facial skeleton. Thus, they are more often fractured due to direct hits during fights. This finding is in accordance with other publications [6, 7, 17, 21, 27]. The lowest MFI rate was observed in the elderly age group, with the main etiology of injuries in this group being falls. Age-related coexisting conditions or comorbidities, such as cardiovascular disease, poor eyesight, osteoporosis, orthostatic collapse, decreased attention to the environment and cognitive decline, were the common reasons for falls in this group of patients. In contrast, falls are the predominant cause of facial fractures in countries where older persons are a growing demographic group in society [4].

The most common MFF site and type following trauma varied among studies. The results from most studies showed that the mandible was the most commonly affected area [6, 7, 9, 15, 17, 20, 21, 26, 27]. However, in this study, the nasal bones were found to be the most common injury site, followed by the mandible and zygomatic complex. The majority of nasal bone fracture incidences were found in cases of RTIs. The reason for this could be the fact that most front row passengers in our country do not wear seat belts. Comparable data presented by other authors [27, 29].

Mandible fractures ranked second among all MFIs in the present study. IV was the most common causative factor of mandible fractures in men, wherein there was no case of IV with female involvement. The most common fracture site was the mandibular angle, followed by the symphysis/parasymphysis and body. Physical alteration tends to result in a higher incidence of angle fracture due to a lateral blow to a mandible; thus, it is considered that the angle is the most often involved site in patients with isolated mandibular fractures, which typically result from assault. The most frequent combined mandible fracture associations in the present study were the angle and the parasymphysis. A similar finding on mandible fracture loci distribution was described in previous studies [19, 23]. In contrast, studies have reported the highest incidence of parasymphysis [9, 15, 24] or condylar fractures [1]. Thus, we supposed that country demographics and level of social development influenced injury etiology and fracture location, respectively.

MFFs can be treated with either closed reduction (conservative) or ORIF (surgical) methods or a combined approach. The decision regarding treatment depends on a variety of factors, such as the nature of the injury, the presence of associated injuries and comorbidities, and the skill of the surgeon. In the present study, close reduction was performed in all patients with nasal bone fractures and patients with minimally displaced zygomatico-maxillary and zygomatic arch fractures. The other fractures were treated by ORIF.

The present study provides information regarding the epidemiology of maxillofacial fractures. This information can be used in healthcare institutions to improve the mechanism for providing urgent and qualified care to patients with MFI. Other government agencies can use these data to implement social education programs to reduce violence and RTA injuries. However, there are some limitations of the study that must be taken into consideration. This article summarizes the results of the epidemiological analysis of one medical center; a multicenter observation could provide more reliable information on the epidemiology of maxillofacial injuries in Armenia. The present study is retrospective; thus, some data in historical cases of patients could be incomplete or not sufficiently detailed to be recorded. The probability of the patient’s concealment of facts about the causes of injury to avoid legal implications was also not excluded.

## Conclusions

Interpersonal violence, followed by RTAs and falls, was the most common cause of MFIs. Males in the 21–30 years age group had the highest MFI incidence rate. The nasal bone was the most common injury site, followed by the mandible and zygomatic complex. Social education with the objective of reducing aggression and interpersonal conflict should be improved, and appropriate RTA prevention strategies should be strengthened and implemented.

## Data Availability

The data that support the findings of this study are available from the corresponding author upon request.

## Abbreviations

MFF: Maxillofacial fracture
MFI: Maxillofacial injury
RTA: Road traffic accident
IV: Interpersonal violence
ORIF: Open reduction internal fixation
MVA: Motor vehicle accident
